# Nutrient additions to seagrass seed planting improve seedling emergence and growth

**DOI:** 10.3389/fpls.2022.1013222

**Published:** 2022-11-23

**Authors:** R.K.F. Unsworth, S.C. Rees, C.M. Bertelli, N.E. Esteban, E.J. Furness, B. Walter

**Affiliations:** ^1^ Seagrass Ecosystem Research Group, Swansea University, Swansea, United Kingdom; ^2^ Project Seagrass, The Yard, Cardiff, Wales, Bridgend, United Kingdom

**Keywords:** *Zostera*, nature-based solution (NBS), marine, eelgrass, microbiome

## Abstract

To maximize the opportunities of seagrass as a nature-based solution requires restoration to occur on a large scale. New methods and knowledge are required that can solve ecological bottlenecks, improving its reliability and effectiveness. Although there is increasing interest in the use of seeds for seagrass restoration there exists a limited understanding of how best to plant them with the most knowledge on germination and seedling emergence coming from laboratory studies. Here we present the results of a novel field study on the emergence success of seeds of the seagrass *Zostera marina* when subjected to varied planting treatments. Seeds were planted into hessian bags according to a factorial design of three treatments (sediment type, detritus addition, and nutrient addition). By adding nutrients to natural sediment, the present study provides some evidence of seagrass shoot emergence and maximum shoot length doubling. The present study provides evidence that even in heavily nutrient-rich environments, seagrass sediments may require additional nutrients to improve seedling emergence and growth. It also highlights the highly variable nature of planting seagrass seeds in shallow coastal environments. Critically this study provides increasing levels of evidence that small subtleties in the method can have large consequences for seagrass restoration and that for restoration to scale to levels that are relevant for nature-based solutions there remain many unknowns that require consideration.

## Introduction

Seagrass restoration is increasingly recognized as a means of creating nature-based solutions to a changing climate, whilst also improving biodiversity, increasing coastal nutrient cycling and supporting wellbeing (Unsworth et al., 2022). Global loss of seagrass has resulted in large areas of soft sediment marine habitat creating new opportunities for restoration. With an increasing understanding of the environmental window for seagrass growth, extensive habitat suitability modelling is taking place to propose targeted areas now potentially suitable for seagrass restoration (Van Der Heide et al., 2009). To maximize these opportunities at large scale, new methods and knowledge are required that can solve ecological bottlenecks (Unsworth et al., 2019). This is particularly relevant as only 37% of published seagrass restoration trials were recorded to be successful 3 years after planting (van Katwijk et al., 2015). This figure is likely much lower given the propensity of academic papers to favour positive restoration results. Across varied environments, we need to understand what methods are suitable for upscaling and the relative costs and benefits of using such approaches, particularly in the context of factors that become bottlenecks to successful restoration such as negative feedbacks.

The high seed production by some species of seagrass at densities of over 1000 seeds^m-2^ (Greve et al., 2005; Jarvis and Moore, 2010) creates a huge opportunity for seagrass restoration, as the production is thought to largely be underutilized and lost to deeper waters. The use of seagrass seeds for restoration is becoming a far more accepted method of conducting this work (Van Katwijk et al., 2016) with projects in the Chesapeake Bay showing project success over scales of thousands of hectares (Orth et al., 2020). When seeds are in high abundance they create a means of preserving and improving genetic diversity within projects and reducing impacts upon donor populations (Reynolds et al., 2013).

The challenge with the use of seeds in restoration projects is that germination is poor, seed loss is high and seedling survival often low (Orth et al., 2006; Eriander et al., 2016; Infantes et al., 2016). Germination can often be lower than 5% (Orth et al., 2006) and seedlings can be highly vulnerable to negative feedbacks in their early development (Maxwell et al., 2017). Identifying the factors limiting the seed to seedling transition is a critical step to understanding seagrasses population dynamics and developing seed-based restoration techniques (Wang et al., 2017). Numerous factors have been proposed as potential cues for seagrass germination and seedling emergence (Orth et al., 2000), with sediment anoxia and low salinity two of the most common triggers recorded to improve success rate (Probert and Brenchley, 1999; Orth et al., 2000).

In addition to studies showing anoxia, salinity and temperature as being potential factors influencing seed germination, there is increasing evidence that other factors can come into play, particularly in the context of the emergence of the seedling and seed loss. Seed predation and burial can also be major drivers of seed loss (Infantes et al., 2016), leading some authors to propose the use of burlap/hessian bags to reduce seed loss (Unsworth et al., 2019). Recent studies have also indicated that nutrients and light may also be important triggers of germination and seedling emergence too (Wang et al., 2017; Alexandre et al., 2018). Nutrients are commonly seen as a negative influence on seagrass, however nutrients may also be a limiting factor in some seagrass systems (Powell et al., 1991), particularly within sediments (Udy and Dennison, 1997). But in many environments, restoration is improved by fertilization, lessening nutrient limitations and improving growth of desired species (Macdonnell et al., 2022). Recent studies on the addition of nutrients to experimental seagrass restoration mesocosms indicate that they have a positive effect on seedling emergence rates (Wang et al., 2017; Macdonnell et al., 2022). Sediment type may also impact upon the availability of nutrients, together with the organic content, and potentially the microbial community present (Fahimipour et al., 2017). Recent use of seagrass detritus as a form of nutrient and microbial dosing within seagrass seed planting has shown potential promise in enhancing seedling emergence, however this has received limited investigation (Unsworth et al., 2019).

Although there exists growing knowledge on the triggers of seagrass germination and seedling emergence (Orth et al., 2000; Xu et al., 2021) the majority of this knowledge comes from laboratory/mesocosm controlled experiments with limited field validation. Applying many of the factors determined within the laboratory to real world restoration projects is not always possible, meaning that knowledge development that is happening is not resulting in as much improved restoration success as should be the case. Only a handful of studies exist where seed planting has been conducted over manipulated experimental conditions. Such experiments present in the literature focus on aspects such as seed density, environmental gradients (Orth and Moore, 1983; Infantes et al., 2016) or seasonality (Coolidge Churchill, 1983) rather than manipulation of environmental conditions.

The use of hessian/burlap bags for planting seagrass seeds not only provides a means of controlling feedbacks during seagrass restoration (Unsworth et al., 2019), but also provides a means of bridging the knowledge gaps between laboratory understanding of seagrass growth and that of field-based restoration. This is because the vesicle that the bags create provides a receptacle enabling manipulation of conditions in the field environment.

The present study provided a highly novel field-based investigation into the use of different substrate and nutrient additions commonly utilized in seagrass seed-based restoration to potentially improve seagrass seed germination and survival. The novelty is not the design or the treatments, but the attempt to manipulate these conditions in the field within ‘real-world’ restoration environments. This was done using hessian bags as a vesicle for sediment, seeds and additional treatments (nutrients and seagrass detritus).

## Methods

In December 2018 an experimental seagrass restoration trial was planted in Dale in West Wales to understand the relative effects of different planting media used within seagrass (*Zostera marina*) seed planting. All sites were within Dale Roads within the Milford Haven Waterway, an area observed to have a large tidal range (7.68m) resulting large flushing and be dominated by fine and silt. Dale has an annual seawater temperature range of 8°C-17°C peaking in August and contains one small-isolated patch (5 m^2^) of natural seagrass approximately 50m north of the experimental sites. The nearest large meadow of *Z. marina* meadows exists in Littlewick (Dale and Gelliswick 6.5km from the dale site and commonly referred to as gelliswick) which has been dated back over 100 years (Kay, 1998) but has been observed to be suffering from elevated nutrients and declining density (Jones and Unsworth, 2016; Bertelli et al., 2021).

Viable seagrass seeds (50 in each) (Infantes and Moksnes, 2018) (were placed in hessian bags and exposed to one of 8 different treatments of additional nutrients, sediment types and detrital inoculant (see **Table 1**). Nutrients were added to the bags using Osmocote™ (5 balls), this contained 15% N, 3.9% P, 10% K, 1.2% Mg, 0.45% Fe, 0.06$ Mn, 0.02% B, 0.05% Cu, 0.02% Mo, and 0.015% Zn. 200ml of sediment was also added to each bag, this was either sterile play sand or locally collected marine sand and bags were either inoculated or not with fresh wet seagrass detritus (50ml) collected from the rotting down stage of seed separation. The use of childs play sand provides a clean alterative to natural local marine sand that doesn’t require such excessive licensing to use and is free of any biosecurity hazards. The inclusion of play sand provides a means of adding in a sand without any additional microbiome of nutrients. One replicate (hessian bag) of each of eight different treatments were placed randomly along an 8m transect line with each bag spread 1m apart. Twelve of these transects were established, creating 12 replicates of each treatment. These were spread over 3 plots (of four lines) immediately adjacent to each other so that they cannot be considered independent sites. Each bag contained 50 seeds, meaning 600 seeds were treated to each treatment. Seeds were collected from Porthdinllaen in North Wales during August 2018 and separated within laboratories at Swansea University (during September). During October and November, seeds were stored in recirculating seawater at ambient light and temperatures.

**Table 1:** Treatment designs used in experimental sites in Dale, Pembrokeshire.

Treatment code	Sediment type	Nutrient addition	Organic inoculant	Number of replicates/bags	Number of seeds
1	Sterile	Yes	Yes	12	600
2	Sterile	No	Yes	12	600
3	Natural	Yes	Yes	12	600
4	Natural	No	Yes	12	600
5	Sterile	Yes	No	12	600
6	Sterile	No	No	12	600
7	Natural	Yes	No	12	600
8	Natural	No	No	12	600

The bags were hand planted onto the seabed and attached to thin lines (with labels) to enable relocation of treatments and to prevent any being washed away. The bags remained partially sunk, but not beneath the sediment. These bags were then observed in April and July 2019 when divers counted shoot number on each bag and measured the longest shoot.

In 2019, collections of seagrass tissue (n=3) for nutrient analysis were conducted in adjacent natural seagrass patches (Jones and Unsworth, 2016). This allowed for the analysis of elemental C, N and P. In addition, analysis of sediment pore water was also conducted at the site to understand background concentrations of nutrients. Seven sediment core samples were taken in close proximity to the experimental plots in Dale using 50ml syringes. The tip of the syringes was cut off and injected into the sediment down to 10cm depth. The content collected within sediment cores were centrifuged. The supernatant was taken of the samples and filtered through a 0.2µm filter. Total oxidised nitrogen (TON), ammonium (NH4(low)+(high), and phosphate (PO4(low)3-(high)) was quantified using a Seal Analytical Continuous Flow system (AA3; SEAL Analytical, Norderstedt, Germany) following the methods of Grasshoff et al. (1983).

Statistical analyses were conducted in PRIMER v7 (Clarke and Gorley, 2006). Data were transformed (square root) where appropriate for count data, to reduce variance of heterogeneity. Univariate analysis consisted of a three-factor (nutrient, detritus and sediment) permutational multivariate analysis of variance (PERMANOVA+; Anderson, 2017) using a Euclidean resemblance matrix to test for differences in relative abundance and shoot length between treatments (**Table 1**).

## Results

In April 2019, seagrass tissue within adjacent patches was recorded to contain 3.5% gDW-1(high) nitrogen and 0.43% g DW-1(high) phosphorus. These values exceed the average values of seagrass throughout the UK (N = 3.58 ± 0.95, P = 0.21 ± 0.07 ^% gDW-1^) (Jones and Unsworth, 2016), indicating the nutrient rich environment of the site. Porewater samples taken around the site in Dale contain 280 ± 15µmoll-1 TON, 25 ± 0.2µmoll^-1^ ammonium and 30 ± 0.1µmol l-1 (high) phosphate.

In April 2019, 86 of the 96 bags could be observed and reliably quantified. Of these 86, 32 contained seagrass (37%) equating to an average density of 1.11± 2.13 shoots per bag and longest leaf of 1.34 ± 2.01mm see (**Scheme 1**). By July 2019, 84 of the 96 bags could be observed and reliably quantified with 40 of these showing seagrass shoots (48%). These shoots had a density of 1.43± 2.37 shoots per bag and longest leaf of 6.38 ± 9.66mm.

**Figure 1:**
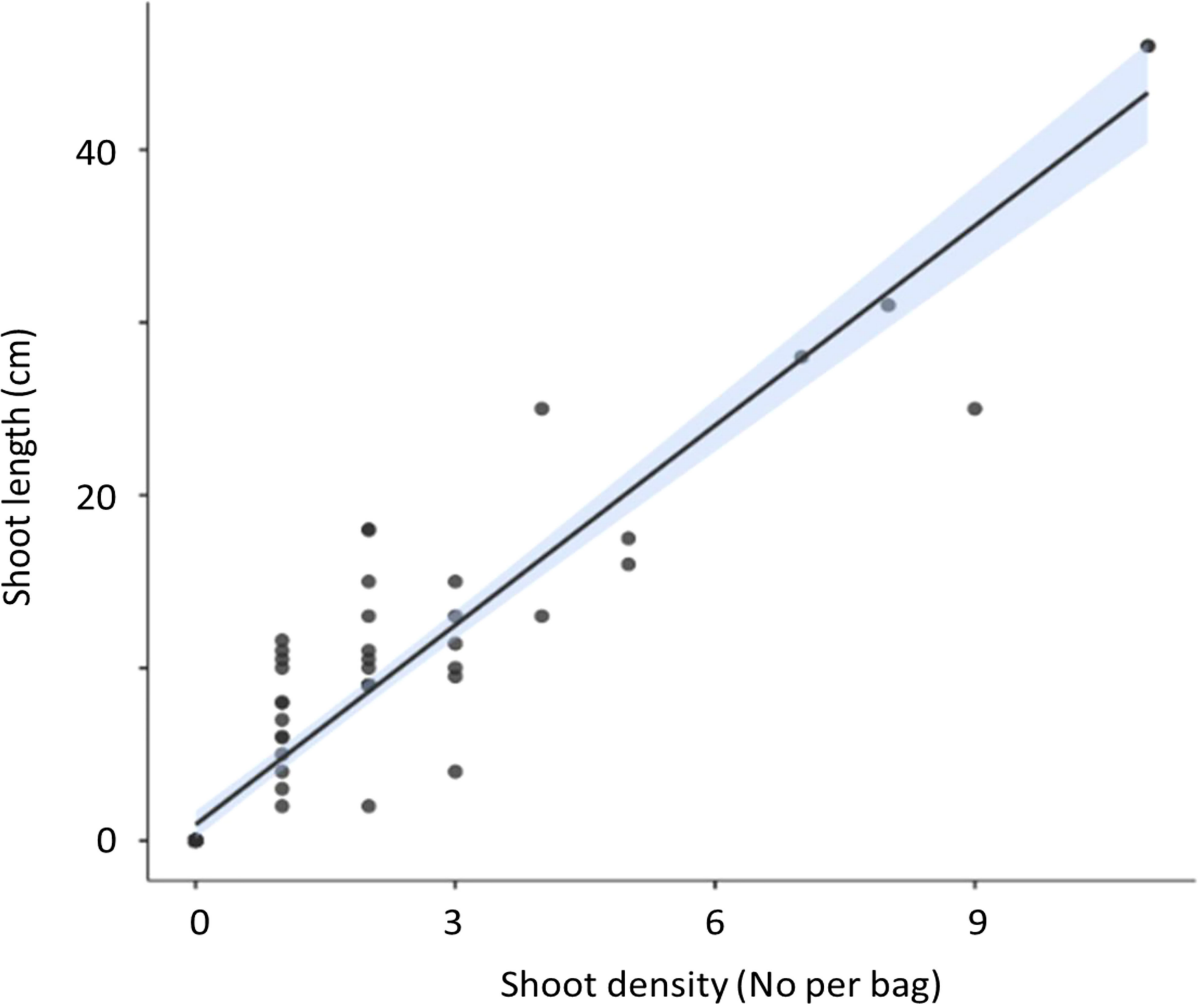
Correlation (± 95% CI) between seagrass shoot density and maximum shoot length of the seagrass *Zostera marina (Z. marina)* in hessian bags in Dale, Wales following experimental planting. Seagrass seeds were planted into hessian bags according to a factorial design of three treatments (sediment type, detritus addition, and nutrient addition).

Shoot density was found to directly correlate with increasing maximum shoot length in both July and April (**Figure 1**).

**Figure 2:**
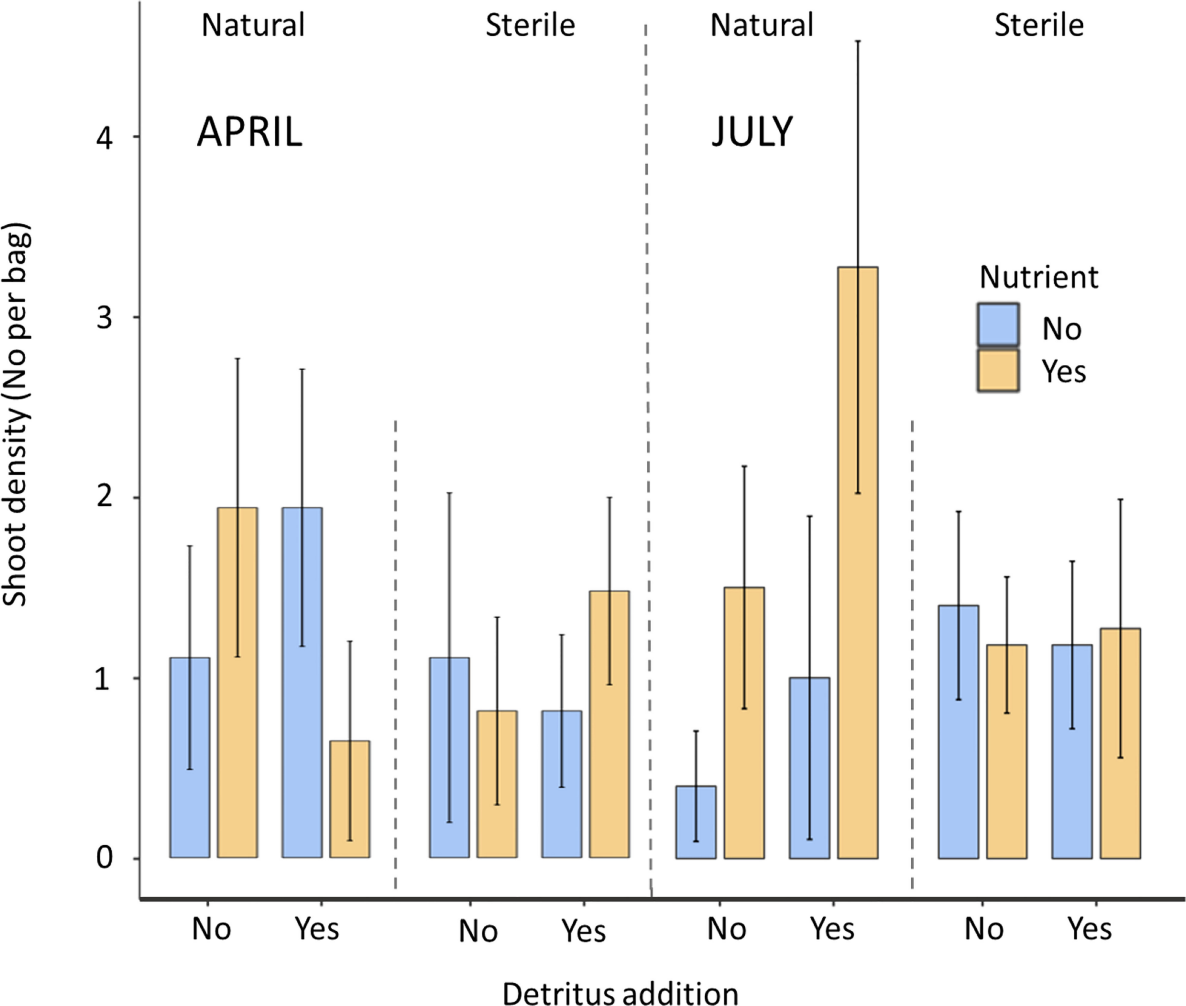
Mean ( ± SD) shoot density of the seagrass *Zostera marina* in hessian bags in Dale, Wales following experimental planting. Seagrass seeds were planted into hessian bags according to a factorial design of three treatments (sediment type, detritus addition, nutrient addition).

**Figure 3:**
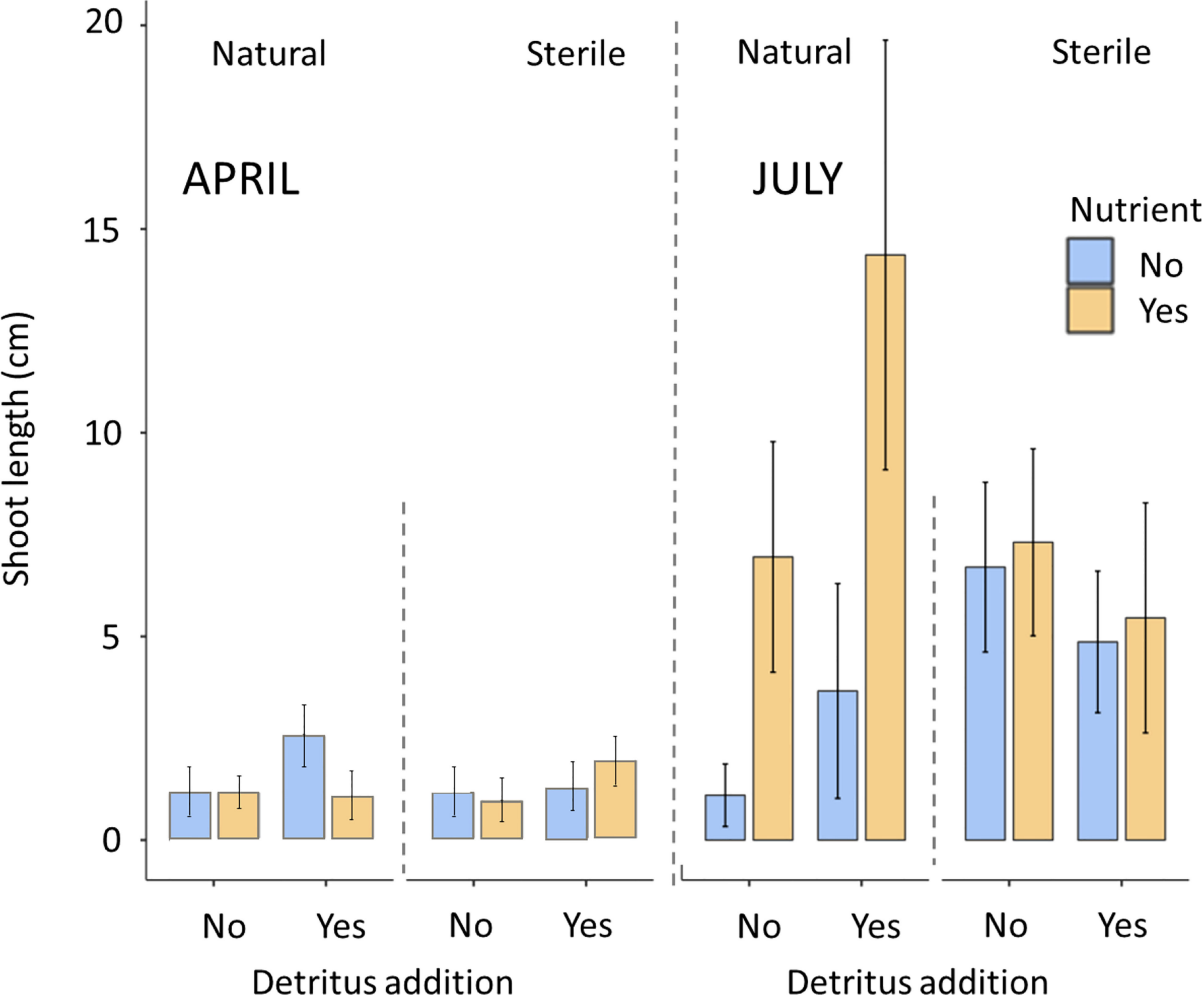
Maximum shoot length (mean ± SD) of seagrass (*Zostera marina*) recorded in hessian bags in Dale, Wales following experimental planting. Seagrass seeds were planted into hessian bags according to a factorial design of three treatments (sediment type, detritus addition, nutrient addition).

In April there was limited differentiation between the density of shoots and the leaf length with respect to the treatments. No significant effects of any of the treatments on seagrass shoot density (P>0.05) or shoot length (P>0.05) were recorded (**Figures 2**, **3**).

By July there were some differences in density and shoot length present between treatments with 44% of natural sediment bags contained seedlings whilst this was higher at 52% for sterile sediment.

66% of bags containing natural sediment together with additional nutrients contained seagrass shoots. This increased to 72% of bags containing shoots with the addition of detritus. In contrast, natural sediment without any additions had the lowest emergence rates of shoots at 20%. All sterile treatments were consistently between 45 and 54%. In July, mean shoot density ranged from 3.27 to 4.15 in the natural, nutrient and detritus treatment compared to 0.4 to 0.97 in the natural no detritus and no nutrient treatment. These differences between treatments were found to be significant with respect to nutrient additions on both seagrass shoot density (P<0.05, F_1,77 =_ 4.33) and longest leaf length (P<0.05, F_1,77 =_ 4.35), both of these parameters had significant interactions with sediment (P<0.05). Detritus addition and sediment type did not significantly affect seagrass shoot density or length (**Figures 2**, **3**).

The highest maximum shoot density and shoot length values across the individual bags (July) were mostly recorded in treatments containing nutrient and detritus additions. The lowest maximum values (excluding zeros) were recorded in sterile sediments without additions.

## Discussion

The present study provides a novel field-based insight into the planting of seagrass seeds into the natural environment and the role of different sediment types and supplements in the emergence of seedlings. We found that in a water body characterised by elevated nutrients and potential areas of eutrophication (Jones and Unsworth, 2016; Bertelli et al., 2021), seagrass seed emergence was still enhanced by nutrient additions into the sediment when planting. This indicates that sedimentary pore waters may not have had optimal nutrients available for seagrass growth. By adding nutrients, the present study provides evidence of seagrass shoot emergence increasing ≈2 fold and maximum shoot length also doubling, however this result only happens in the presence of natural sediment. Others have recorded planted seagrass to benefit from the addition of nutrients (Wang et al., 2017; Macdonnell et al., 2022), with nutrients outweighing the influence of light cues (Wang et al., 2017) and the slow release of nutrients also found to be beneficial (Macdonnell et al., 2022), but this is the first field study to demonstrate this with seeds.

In tropical calcareous environments typical of low nutrient conditions, sediment nutrient additions have been recorded to enhance seagrass growth (Udy et al., 1999), this is typically considered to be due to phosphorus limitation (Short, 1987). However, conditions within the present study are those of terrigenous sediments where phosphorus limitation is unlikely. In line with the results of the present study, some authors have demonstrated terrigenous sediments to be replete in nitrogen (Short, 1987), even though the associated water body is enriched with elevated nitrogen.

Enhanced seagrass growth with additional nitrogen provision to the sediments was not ubiquitous across the treatments and had a significant interaction with sediment type. This positive effect was only observed within natural sediments rather than sterile ones. The reason for this lack of response in sterile sediment (play sand) is unclear, however we suggest two potential hypotheses, high permeability of the play sand not maintaining the additional nutrients, or the role of a natural microbiome helping facilitate nutrient uptake in natural sediments. The play sand used here contained no organics, as it provides a means of ensuring no alien or invasive species are spread with seed planting. The origin of this sand is that it’s ‘child’s play sand’ with no builders additives. As a result of its origin the particle sizes are far more consistent than natural sediment, increasing permeability due to the lack of fines. We hypothesize that this permeability may lead to the loss and dilution of nutrients from adjacent background porewater.

Although we have as yet limited knowledge of how the sediment and rhizosphere microbiome is influencing seagrass function, there is clear data in the literature showing a distinct microbiome on the roots of *Z. marina* relative to surrounding environments (Fahimipour et al., 2017). The taxa of the microbiome has a high dominance of organisms thought to have functional roles in nutrient cycling. Although we don’t have any cause and effect here we do know that the natural sediment came from a nearby location within 50m of extensive seagrass that may have inoculated seedlings early on potentially providing a microbiome that could utilize more effectively the nutrient additions.

This study is marked by high levels of variability between and within treatments, leading to only marginal statistical differences between treatments. This variability is in contrast to the majority of laboratory/mesocosm experiments on seed planting and germination where external influences are controlled in a manner not possible *in situ*. Many lab studies have investigated seagrass seed burial depth as a key determinant of success and a depth of 2cm appears to be broadly consistent across studies (Marion and Orth, 2012; Xu et al., 2021). Seeds planted in bags aren’t at a controlled depth even though bags were planted in a controlled manner, this is because upon placement in the bag whilst in the lab, mixing and disturbance happens on route, adding an unquantified level of variability into the design.

Previous methodological development work had included seagrass detritus as a means of inoculating the microbiological environment of the seagrass seeds to assist with seedling development and survival (Unsworth et al., 2019). In the present study, addition of detritus as a potential stimulant of the microbiome did not show any influence on the success of seeds and their emergence.

In conclusion, this present study provides evidence that even in heavily nutrient rich environments, seagrass sediments may require additional nutrients to improve seedling emergence and growth. It also highlights the highly variable nature of planting seagrass in shallow coastal environments. Critically this study provides increasing levels of evidence that small subtleties in method can have large consequences for seagrass restoration and that for restoration to scale to levels that are relevant for nature-based solutions there remain many unknowns. We find that small changes to the sediment surrounding seeds may have a significant impact upon seedling emergence and success.

## Data availability statement

The raw data supporting the conclusions of this article will be made available by the authors, without undue reservation.

## Author contributions

RU undertook all elements of the project. SR and CB helped design the experiment. All authors undertook the experiment and commented on the manuscript.

## Funding

This study was supported by the UK Government Research Funding: NERC RESOW project NE/V016385/1 and the Welsh Government ERDF Funding: SEACAMS.

## Conflict of interest

The authors declare that the research was conducted in the absence of any commercial or financial relationships that could be construed as a potential conflict of interest.

## Publisher’s note

All claims expressed in this article are solely those of the authors and do not necessarily represent those of their affiliated organizations, or those of the publisher, the editors and the reviewers. Any product that may be evaluated in this article, or claim that may be made by its manufacturer, is not guaranteed or endorsed by the publisher.
